# Evidence of validity of the Stress-Producing Life Events (SPLE) instrument

**DOI:** 10.11606/S1518-8787.2018052000173

**Published:** 2018-02-07

**Authors:** Marta Rizzini, Alcione Miranda dos Santos, Antônio Augusto Moura da Silva

**Affiliations:** I Universidade Federal do Maranhão. Programa de Pós-Graduação em Saúde Coletiva. São Luís, MA, Brasil; II Universidade Federal do Maranhão. Departamento de Saúde Pública. São Luís, MA, Brasil

**Keywords:** Pregnant Women, Psychological Stress, Life Change Events, Scales, Validation Studies, Gestantes, Estresse Psicológico, Acontecimentos que mudam a vida, Escalas, Estudos de Validação

## Abstract

**OBJECTIVE:**

Evaluate the construct validity of a list of eight Stressful Life Events in pregnant women.

**METHODS:**

A cross-sectional study was conducted with 1,446 pregnant women in São Luís, MA, and 1,364 pregnant women in Ribeirão Preto, SP (BRISA cohort), from February 2010 to June 2011. In the exploratory factorial analysis, the promax oblique rotation was used and for the calculation of the internal consistency, we used the compound reliability. The construct validity was determined by means of the confirmatory factorial analysis with the method of estimation of weighted least squares adjusted by the mean and variance.

**RESULTS:**

The model with the best fit in the exploratory analysis was the one that retained three factors with a cumulative variance of 61.1%. The one-factor model did not obtain a good fit in both samples in the confirmatory analysis. The three-factor model called Stress-Producing Life Events presented a good fit (RMSEA < 0.05; CFI/TLI > 0.90) for both samples.

**CONCLUSIONS:**

The Stress-Producing Life Events constitute a second order construct with three dimensions related to health, personal and financial aspects and violence. This study found evidence that confirms the construct validity of a list of stressor events, entitled Stress-Producing Life Events Inventory.

## RESUMO

**OBJETIVO:**

Avaliar a validade de construto de uma lista com oito Eventos de Vida Estressantes em gestantes.

**MÉTODOS:**

Foi conduzido um estudo transversal com 1.446 gestantes em São Luís, MA, e 1.364 em Ribeirão Preto, SP (coorte BRISA), de fevereiro de 2010 a junho de 2011. Na análise fatorial exploratória, utilizou-se a rotação oblíqua promax e para o cálculo da consistência interna, a confiabilidade composta. A validade de construto foi determinada por meio da análise fatorial confirmatória com o método de estimação dos mínimos quadrados ponderados ajustados pela média e variância.

**RESULTADOS:**

O modelo com o melhor ajuste na análise exploratória foi o que reteve três fatores com uma variância acumulada de 61,1%. O modelo com um fator não obteve um bom ajuste em ambas as amostras na análise confirmatória. O modelo com três fatores denominado Eventos de Vida Produtores de Estresse apresentou um bom ajuste (RMSEA < 0,05; CFI/TLI > 0,90) para as duas amostras.

**CONCLUSÕES:**

Os Eventos de Vida Produtores de Estresse constituem um construto de segunda ordem com três dimensões relacionadas à saúde, aos aspectos pessoais e financeiros e à violência. Este estudo encontrou evidências que confirmam a validade de construto de uma lista de eventos estressores, intitulado Inventário de Eventos de Vida Produtores de Estresse.

## INTRODUCTION

Stress is very common in pregnancy because it is a period of intense changes in the woman’s life from the psychological, social and physical point of view^1^ ^,^ ^2^. Stress-related pregnancy complications represent an important cause of maternal and perinatal morbidity and mortality^1^.

Stress during pregnancy can be studied through the evaluation of Life Events (LE), which are vital experiences of physical or psychological characteristics. They may represent significant or discrete changes in this life span. Life Events, when investigated from the perspective of stress, are called Stressful Life Events (SLE)^3^.

Despite the constant debates about the methods of measurement of the SLE (checklist/scale or interview), the standardized list of stressors continues being the most used approach for the evaluation of stress in epidemiological studies, for its ease of application and low cost^4^.

There are many checklists developed and validated in several countries to measure SLE. Most are made up of long lists of events, some surpassing 100 stressful events^5^. However, these lists are often used in researches with modifications to meet the characteristics of the sample and decrease the number of items, which greatly increases the time of application^6^ ^,^ ^7^. Thus, many studies choose SLE from several checklists without providing information about their psychometric properties^3^ ^,^ ^8^ ^,^ ^9^. Most of these lists were created in developed countries between the 1960s and 1990s^10^, especially those most used today when the stressors had different characteristics and intensities^11–14^.

SLE are used to measure stress with some of these checklists also in Brazil, but with modifications or simple translations of scales validated in other languages^15^ ^,^ ^16^. There is an important gap in assessing the validity of these scales that measure SLE in Brazil.

There is a question of whether SLE constitute a construct because of the impossibility of grouping them into dimensions and forming a construct^3^. There are problems with event memory and reliability in checklist methods that measure SLE. Considering that SLE measures are not reliable or are invalid, these deficiencies could attenuate their association with health outcomes or make these relationships difficult to interpret^17^.

Researchers from the Instituto de Medicina Social of the Universidade do Estado do Rio de Janeiro grouped eight SLE through closed-ended questions, with dichotomous answers, covering events that occurred in the previous 12 months^18^. The authors opted for the use of simple and brief questions, a self-administered instrument of easy filling. These eight SLE were called Stress-Producing Life Events (SPLE).

However, for stressful situations to be measured reliably, it is necessary to ensure valid instruments to measure the SPLE. The approach through exploratory and confirmatory analysis is considered an appropriate statistical tool to obtain validity, identifying if the SPLE form a construct. Therefore, this study aimed to evaluate the construct validity of the eight SPLE items in Brazilian pregnant women.

## METHODS

This psychometric study is linked to the cohort called “Etiologic Factors of Preterm Birth and Consequences of Perinatal Factors in Child Health: birth cohorts in two Brazilian cities”^19^. This project, called BRISA, was developed by the Graduate Program in Collective Health of the Universidade Federal do Maranhão and by the Faculdade de Medicina de Ribeirão Preto.

These are convenience samples due to the lack of records of pregnant women or women who are prenatal in São Luís, state of Maranhão, and Ribeirão Preto, state of São Paulo, and it is not possible to obtain a representative sample. The pregnant women were recruited in the main public and private maternity hospitals, being registered to be interviewed from the 22nd to the 25th week of gestation. The women were only included in the study if they had undergone the first ultrasound examination with less than 20 weeks of gestation and if they had the intention to have the childbirth in one of the maternity wards of the municipality where the study was being carried out. Women with multiple pregnancies were not included in the study.

A total of 1,447 pregnant women were recruited from February 2010 to June 2011 in São Luís. The final sample for analysis was 1,446 participants after the exclusion of a pregnant woman for not completing the inventory used in this research. In Ribeirão Preto, data were collected from February 2010 to February 2011. The sample consisted of 1,400 pregnant women. Data from 1,364 women were used since 36 did not complete the information on SLE.

For the data collection of the stressor events, eight items with SPLE reports were used, according to the procedure described by Lopes and Faerstein^18^, included in the Self-Applied Prenatal Questionnaire of the BRISA project. The items were surveyed using a list with dichotomous responses (yes, no), to measure the number of stressors in the last 12 months. The items covered: a health problem that resulted in the interruption of usual activities for more than one month; hospital admission due to illness or accident; death of next of kin; severe financial difficulties; forced relocation of the housing; separation or divorce; physical aggression; and theft or robbery. Despite the existence of severity scales for the evaluation of stressful events, studies prioritize the use of direct and simple questions and evaluate the role of the occurrence of more than one event by the score related to the number of events^20^.

Descriptive analyses of the two samples were performed, in which absolute and relative frequencies were estimated. The frequency of positive responses to the presence of stressful events using the test for proportions (p < 0.05) was verified to determine statistically significant differences between the two samples.

The study was carried out in two stages. The data from São Luís and later from Ribeirão Preto were evaluated, with the objective of verifying the stability of the factorial solution in two separate groups. The total samples for São Luís (n = 1,446) and Ribeirão Preto (n = 1,364) were used in the exploratory and confirmatory analyzes with the same ones independently analyzed, following the same steps and procedures.

In the exploration of the data, we used the Exploratory Factorial Analysis (EFA), with a factor extraction method, the robust weighted least squares estimator (WLSMV) for use with categorical variables. In order to determine the number of factors to be retained, the eigenvalues > 1 were considered and the cumulative variance criterion was used, in which the factor extraction is continued until reaching the level of 60% of the variance. The objective is to identify the minimum number of factors that maximize the number of total variance explained^21^ ^,^ ^22^. To facilitate the interpretation of the results, the promax oblique factorial rotation was used, since it allows the factors to be correlated with each other^23^.

In the final factorial structure of the EFA, we verified the existence of similar factorial loads in two or more factors in the same item, with the difference between the absolute values of the loads smaller than 0.10^23^. Factorial loads were considered significant when above 0.30, the minimum value needed for the variable to be a useful representative of the factor^23^.

In the Factorial Confirmatory Analysis (FCA), the construct validity was evaluated by testing three models. The one-dimensional model (model 1) consisted of the eight observed variables (items 1 to 8). Models 2 and 3 were tested with three latent factors as a result of the EFA (first order factorial structure) and a second-order factor to determine if the three latent dimensions formed the SPLE construct. Model 3 was generated from the Modification Indexes (MI) that suggest modifications in relation to the initial hypothesis. The estimation method used was the same one previously mentioned (WLSMV), recommended for the analysis of categorical variables and also the matrix of tetrachoric correlation in the case of binary data^24^ ^,^ ^25^.

The following goodness of fit indices were used: a) p < 0.05 and upper limit of the 90% confidence interval < 0.08 for RMSEA (Root Mean Square Error of Approximation)^25^; b) values higher than 0.90 for the Comparative Fit Index (CFI) and Tucker-Lewis Index (TLI); c) value less than 1 for the WRMR (Weighted Root Mean Square Residual) index^24^ ^,^ ^25^. The chi-squared, degrees of freedom and p-value were evaluated but were not adopted as parameters for the fit of the model, due to its sensitivity to sample size.

In sequence, the internal consistency for models 2 and 3 was evaluated through composite reliability, in which values ≥ 0.70 are considered satisfactory^24^. The correlations between the factors were evaluated and, according to the literature recommendation, the values > 0.85 were considered as suggestive of absence of discriminant factorial validity^23^.

The data were entered in Stata 11.0 statistical package. For the exploratory and confirmatory analyses, the statistical package Mplus, version 7.0 (Muthén & Muthén, Los Angeles, USA) was used.

This study was approved by the ethics committees of the Hospital Universitário da Universidade Federal do Maranhão (Protocol 4771/2008-30) and the University Hospital of the Faculdade de Medicina de Ribeirão Preto (Protocol 4116/2008). The pregnant women signed a free and informed consent form. An adult companion also signed the term for those under 18 years of age.

## RESULTS

In the sample of São Luís (n = 1,446), the mean age was 25.7 years (SD = 5.5) and 75.4% had completed high school. In class C, there were 67.7%, followed by classes D/E with 16.4% and A/B with 15.9%. In Ribeirão Preto (n = 1,364), the mean age was 25.9 years (SD = 6.9) and 64.7% had a high school education. Class C corresponded to 60.0%, 27.9% to classes A/B and 11.7% to classes D/E.

The items with higher and lower percentages of positive responses to SPLE for São Luís were 4 (37.2%) and 8 (8.0%), respectively. Likewise, in Ribeirão Preto, 4 (37.6%) and 8 (4.9%), respectively. Only items related to financial aspects (items 4 and 5) were not statistically significant in the São Luís and Ribeirão Preto samples in relation to the percentages of positive responses to the presence of stressors (Table 1).

**Table 1:** Items from the Stress-Producing Life Events (SPLE) list and positive response percentages. São Luís, state of Maranhão and Ribeirão Preto, state of São Paulo, 2010 to 2011.

Dimension/Item	Question (in the last 12 months)	SL^a^ (%)	RP^b^ (%)	p^c^
Health				
1	Have you had any health problems that prevented you from performing any of your usual activities (work, study, or leisure) for more than a month?	19.6	15.5	**0.004**
2	Were you hospitalized for one night or more due to illness or accident?	13.5	11.1	**0.046**
Personal and financial				
3	Was there a death of some close relative (father, mother, spouse, companion, child, or sibling)?	23.4	16.9	**< 0.001**
4	Have you faced more severe financial difficulties than usual?	37.2	37.6	0.827
5	Were you forced to move to a new house against your will (for example, due to rent increase)?	13.8	15.4	0.218
6	Have you experienced any breakup in a romantic relationship, including divorce or separation?	19.4	15.5	**0.008**
Violence				
7	Were you attacked or robbed, that is, had your money or any goods taken, through use or threat of violence?	10.8	5.2	**< 0.001**
8	Were you a victim of physical aggression?	8.0	5.0	**0.001**

^a^ São Luís.

^b^ Ribeirão Preto.

^c^ Proportions test.

Values with statistical significance in relation to the presence of stressor events between the two samples are presented in bold.

In order to test whether the SPLE were grouped in dimensions, the Exploratory Factor Analysis (EFA) was performed. In the evaluation of the factorial structure, the model with the best fit was what retained three factors with a cumulative variance of 61.1%. The three-dimensional EFA presented the best fit in São Luís (RMSEA = 0.000; CFI = 1.000; TLI = 1.031) and Ribeirão Preto (RMSEA = 0.000; CFI = 1,000; TLI = 1.020): a) factor (1 and 2) forming the dimension called Health; b) factor 2 (items 3, 4, 5 and 6) representing the Personal and Financial Aspects dimension; c) factor 3 (items 7 and 8) generating the dimension of Violence. All loads were above 0.30 except for item 3 (death of a close relative) in the São Luís sample. This item was initially not excluded because in EFA sample size is considered to identify a factorial load as significant^23^ ^,^ ^25^. The decision on the permanence or exclusion of this item was decided by the FCA, in which it showed that the preservation of the item improved the fit of the model.

**Figure 1:**
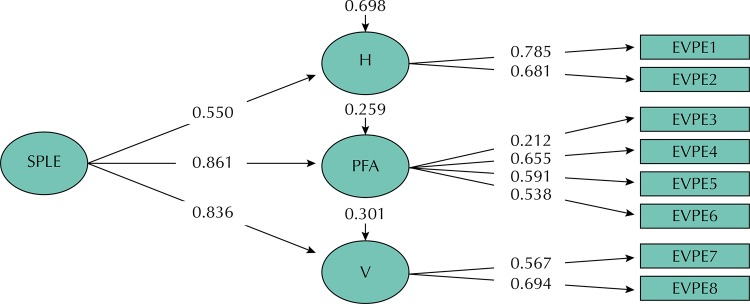
Confirmatory factorial analysis of the three-dimensional model (model 2) for pregnant women. São Luís, state of Maranhão, 2010 to 2011.

SPLE: Stress-Producing Life Events; H: Health; PFA: personal and financial aspects; V: violence; EVPE 1 to 8: items from the Stress-Producing Life Events Inventory

**Figure 2:**
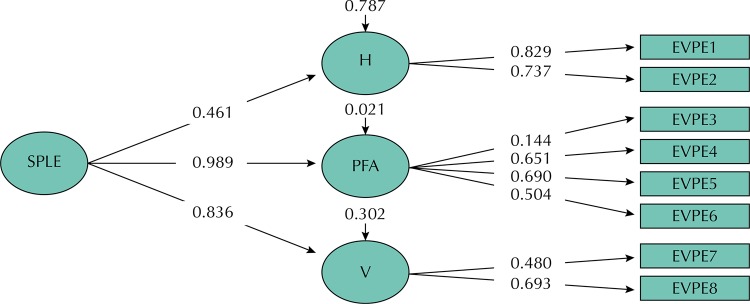
Confirmatory factorial analysis of the three-dimensional model (model 2) for pregnant women. Ribeirão Preto, state of São Paulo, 2010 to 2011. SPLE: Stress-Producing Life Events; H: Health; PFA: personal and financial aspects; V: violence; EVPE 1 to 8: items from the Stress-Producing Life Events Inventory

In the Factorial Confirmatory Analysis (FCA), three models were tested to assess whether the eight items of stressors actually measured the SPLE construct. The one-dimensional model (Model 1) did not obtain a good fit in São Luís and Ribeirão Preto (Table 2). The model generated from the grouping of items revealed in the three-dimensional EFA (Model 2) presented the best fit in São Luís (RMSEA = 0.023, CFI = 0.978, TLI = 0.963) (Table 2 and Figure 1) and Ribeirão Preto (RMSEA = 0.033, CFI = 0.956, TLI = 0.927) (Table 2 and Figure 2).

**Table 2:** Fit indexes for the model with one factor (model 1) and models with three factors (models 2 and 3). São Luís, state of Maranhão and Ribeirão Preto, state of São Paulo, 2010 to 2011.

Indexes	Model 1^a^		Model 2^b^		Model 3^c^	
		
	São Luís	Ribeirão Preto	São Luís	Ribeirão Preto	São Luís	Ribeirão Preto
χ^2d^	120,538	124,037	29,731	42,706	13,888	36,590
Degrees of freedom	20	20	17	17	16	16
p	< 0.001	< 0.001	0.0284	0.0005	0.6070	0.0024
RMSEA^e^	0.054	0.062	0.023	0.033	< 0.001	0.031
90%CI	0.044–0.064	0.052–0.072	0.007–0.036	0.021–0.046	0.000–0.021	0.018–0.044
P	0.257	0.029	0.999	0.987	0.999	0.993
CFI^f^	0.855	0.821	0.978	0.956	1.000	0.964
TLI^g^	0.796	0.749	0.963	0.927	1.000	0.938
WRMR^h^	1.538	1.728	0.803	0.988	0.538	0.910

^a^ Model with one factor.

^b^ Model with three factors suggested by the Exploratory Factor Analysis.

^c^ Model with item 6 simultaneously loading in the personal/financial aspects and violence dimensions.

^d^ Chi-squared test.

^e^ Root Mean Square Error of Approximation.

^f^ Comparative Fit Index.

^g^ Tucker-Lewis Index.

^h^ Weighted Root Mean Square Residual.

The standardized estimates of the factorial loads of the observed variables (instrument items) were above 0.50, except for the load referring to item 3 for São Luís (0.212) and items 3 (0.144) and 7 (0.480) for Ribeirão Preto, however, all were significant (p < 0.001). When the three dimensions formed the SPLE construct, the loads remained above 0.7, except for the Health factor for the São Luís (0.550) and Ribeirão Preto (0,461) samples (Table 3).

**Table 3:** Standard factor loads of confirmatory factor analysis, reliability estimates, and correlation between factors for the three-dimensional models 2 and 3. São Luís, state of Maranhão and Ribeirão Preto, state of São Paulo, 2010 to 2011.

Dimensions	Model 2		Model 3	
	
	Factorial loads; p		Factorial loads; p	
	
	São Luís	Ribeirão Preto	São Luís	Ribeirão Preto
Health				
1.Health problems	0.785; < 0.001	0.829; < 0.001	0.785; < 0.001	0.829; < 0.001
2.Hospitalization	0.681; < 0.001	0.737; < 0.001	0.681; < 0.001	0.737; < 0.001
Personal and financial aspects				
3.Death of close relative	0.212; < 0.001	0.144; < 0.001	0.222; < 0.001	0.146; < 0.001
4.Severe financial difficulties	0.655; < 0.001	0.651; < 0.001	0.731; < 0.001	0.675; < 0.001
5.Forced change of residence	0.591; < 0.001	0.690; < 0.001	0.618; < 0.001	0.713; < 0.001
6.Divorce/Separation	0.538; < 0.001	0.504; < 0.001	0.184; 0.063	0.081; 0.750
Violence				
6.Divorce/Separation	-	-	0.425; < 0.001	0.501; 0.053
7.Violência	0.567; <0.001	0.480; < 0.001	0.561; < 0.001	0.480; < 0.001
8.Physical agression	0.694; < 0.001	0.693; < 0.001	0.713; < 0.001	0.699; < 0.001
SPLE construct				
Health	0.550; < 0.001	0.461; < 0.001	0.589; < 0.001	0.483; < 0.001
Personal and financial aspects	0.861; < 0.001	0.989; < 0.001	0.734; < 0.001	0.918; < 0.001
Violence	0.836; < 0.001	0.836; < 0.001	0.756; < 0.001	0.782; < 0.001
Compound reliability				
Health	0.70	0.76	0.70	0.76
Personal and financial aspects	0.58	0.58	0.48	0.49
Violence	0.57	0.52	0.63	0.59
Correlation between factors				
Ø (f1 ↔ f2)	0.473	0.456	0.432	0.444
*Ø* (f1 ↔ f3)	0.460	0.385	0.445	0.378
*Ø* (f2 ↔ f3)	0.720	0.827	0.555	0.719

SPLE: Stress-Producing Life Events

The compound reliability for the São Luís sample was 0.70, 0.58 and 0.57 for health, personal and financial aspects and violence, respectively. In Ribeirão Preto, the compound reliability was 0.76, 0.58 and 0.52, respectively (Table 3). The discriminant validity was satisfactory for both samples.

The fit of Model 3 was good for the two cities. In this, the suggested modification with the highest value (MI = 16,092) was incorporated with item 6, simultaneously being part of the dimensions of personal and financial aspects and violence. However, the factorial loads of this item did not present statistical significance for São Luís (0.063) and Ribeirão Preto (0.750) and was not considered theoretically plausible (Table 3).

## DISCUSSION

The SPLE applied in two Brazilian cities presented evidence of constituting a construct of second order. This allowed them to be objectively measured in pregnant women. These were called Stress-Producing Life Events Inventory (SPLEI). The SPLEI presented good psychometric quality, forming a structure with dimensions called health, personal/financial aspects and violence, an arrangement identified in the EFA and corroborated in the FCA.

One limitation of this study is the use of a convenience sample that restricts the external validity of the results. However, most of the validation studies are performed with convenience samples and in the most commonly used SLE checklists validated internationally, none were performed in a representative sample^11–14^. The SLEs examined reflected experiences in the previous 12 months, a limitation in SLE studies. However, because of the magnitude of the observed events (undesirable or negative life events), this probably did not pose a problem for SPLEI. The memory of major stressful events is less subject to distortions as are evaluations related to mood and emotions^26^.

On the other hand, the sample size was satisfactory to give precision to the estimates and the percentage of missing data was small in São Luís (0.1%) and Ribeirão Preto (2.6%). Another positive point was the evidence of discriminant factorial validity. The most used checklists for SLE measurement were elaborated in developed countries in the previous decades^10^. They did not contain, for example, essential items such as physical aggression and violence, important stressors in developing countries with higher levels of violence. With lifestyle changes, many important stressors were absent from the lists and others with no purpose remained, compromising the results. Therefore, the advantage of the SPLEI is to be a simple, brief and valid tool for pregnant women with relevant and non-specific stressors that encompass major stress-causing events.

The model with the best fit in the evaluation of the factorial structure performed through the EFA was the one that retained three factors with items 1 and 2 in the health dimension (factor 1), item 3 to 6 in the personal and financial aspects dimension (factor 2) and items 7 and 8 in the violence dimension (factor 3). The EFA revealed that item 3 (death of a close relative) had a reduced load (0.218). However, this item was not excluded, because the sample size is also considered in the exploratory analysis to identify a factorial load as significant, i.e., a load between 0.20 and 0.30 is statistically significant in a large sample (> 350)^24^. In the same way, it was verified that the permanence of this item improved the fit of the model in the FCA and the corresponding factor load obtained statistical significance.

Item 6 (“In the past 6 months, have you experienced any breakup in a romantic relationship, including divorce or separation?”) generated similar loads on personal and financial (0.433) and violence (0.430) factors in the EFA. This suggests that the same variable could contribute to the construction of different dimensions. Possibly, this was due to the fact that the termination of amorous relationships (personal and financial aspects dimension) is correlated with physical aggression (violence dimension) between partners in part of the São Luís sample. However, the difference in the loads between the factors was less than 0.10 and a confirmatory perspective was used. The theoretical elements justify the permanence of this item^24^ since the “breakup of a romantic relationship” is considered one of the main stressors for pregnant women^2^ ^,^ ^14^.

The one-dimensional model (model 1) did not present satisfactory fit in both cities in the FCA, reinforcing the results of the exploratory analysis. Some SLE checklists found in the literature were unidimensional but validated by means of correlation coefficients between groups or between instruments, without EFA investigation^11^ ^,^ ^12^. Others were developed with different dimensions^10^ ^,^ ^13^ ^,^ ^14^, however, the number varied according to the sample, subject under study and the number of stressors that constitute the instrument.

The second order three-dimensional model (model 2) obtained the best fit indexes in both cities in the FCA, which showed the validity of the construct that reflects the theoretical construct (SPLE), indicating relevant values. This model presents the dimension called Health with items related to health problems and hospitalization, considered important stressors, especially for pregnant women^13^. The validated and most used scales that measure SLE have questions about the appearance of diseases, whether acute or chronic^10–14^. The second dimension is related to the pregnant woman’s personal (ending of relationship and death of a close relative) and financial (severe financial difficulties and change of residence), always present in SLE checklists for any population^10–14^. The dimension violence – theft or robberies and physical aggression suffered by the pregnant woman – was the one that appeared least on the validated scales. Possibly because a large part was developed between 1967 and 1981, a period in which violence did not have the current importance^10^.

In model 2, only item 3 obtained low factorial loads in both cities (below 0.50), indicating the small direct effect that the latent variable (personal and financial aspects dimension) has on the observable indicator (item 3). In the literature, this is explained as problems of comprehension of content and meaning^23^, but does not apply to a stressful event as marked and punctual as death (“In the last 12 months, was there a death of some close relative – father, mother, spouse, companion, child, or sibling?”).

Reliability obtained better results for the health dimension in São Luís (0.70) and Ribeirão Preto (0.76) since items 1 and 2 are correlated (health problems and hospitalization). In the personal and financial aspects dimension, questions of different types of events that generated smaller correlations (0.58 for both samples) were found. For the violence dimension, reliability was lower in São Luís (0.57) and Ribeirão Preto (0.52), possibly due to problems related to the low frequency of positive responses to the presence of events, generating little variance for these items. The reduced number of items in stressors at scales also decreases reliability^21^ ^,^ ^27^. International studies have reported problems with reliability in a list of stressors, but using the intraobserver reliability indicator^17^ ^,^ ^28^.

A stressful event that is very present in SLE lists and not grouped in the SPLEI is related to the loss of employment of the pregnant woman/partner since, besides the restriction on the financial question, it also generates changes in the routine of individual’s life^11^ ^,^ ^13^. Another relevant event is related to the health problems of family members or friends^12^ ^,^ ^13^. The introduction of items related to “job loss” and “family or friends’ health problems” to make up the financial and health dimension, respectively, would be a welcome step in future studies.

The Modification Indexes (MI) generated model 3 in both samples, suggesting that item 6 could be part of the dimensions of personal and financial aspects and violence, simultaneously. This modification reduced the chi-squared of the model and slightly increased the value of some factorial loads. This, however, is not theoretically plausible, since item 6 refers to a breakup in a romantic relationship (personal and financial aspects dimension), not composing the violence dimension. The factorial loads of this item composing two dimensions did not present statistical significance in both samples.

In conclusion, SPLE formed a second-order construct with dimensions related to health, personal and financial aspects and violence for both cities. Model 2 showed an excellent fit and was structurally valid to measure SPLE in pregnant women. However, it is necessary to test the inclusion of items related to “job loss” and “health problems of family or friends”, important stressors reported in the literature that could increase the reliability of the instrument.

Stressors have a relevant role in the precipitation of mental and somatic disorders and are an interesting subject for clinical and population-based studies. SPLEI is a simple screening tool for stressors in clinical and primary care settings and can be filled by people with low education levels.
